# Storage of *Mycobacterium tuberculosis* culture isolates in Microbank^TM^ beads at a South African laboratory

**DOI:** 10.4102/ajlm.v12i1.2172

**Published:** 2023-10-25

**Authors:** Anura David, Lesley E. Scott, Pedro Da Silva, Elizabeth Mayne, Wendy S. Stevens

**Affiliations:** 1 Wits Diagnostic Innovation Hub, Faculty of Health Sciences, University of the Witwatersrand, Johannesburg, South Africa; 2 National Priority Program, National Health Laboratory Services, Johannesburg, South Africa; 3 Division of Immunology, University of Cape Town, Cape Town, South Africa

**Keywords:** *Mycobacterium tuberculosis*, culture isolates, storage, liquid culture, Microbank beads

## Abstract

**Background:**

*Mycobacterium tuberculosis* complex (MTBC) isolates are typically stored at −70 °C in cryovials containing 1 mL aliquots of a liquid medium, with or without 50% glycerol. Multiple uses of the culture stock may decrease the strain viability while increasing the risk of culture contamination. Small culture aliquots may be more practical; however, storage capacity remains challenging. Microbank^TM^ beads (25 beads/vial) for the long-term storage of fungal cultures is well documented, but their use for storing MTBC isolates is uninvestigated.

**Objective:**

The study aimed to determine the feasibility of using Microbank^TM^ beads for long-term storage of MTBC isolates at a laboratory in South Africa.

**Methods:**

In February 2020, 20 isolates in liquid culture were stored in Microbank^TM^ beads, following an in-house developed protocol, at −70 °C. At defined time points (16 months [15 June 2021] and 21 months [18 November 2021]), two beads were retrieved from each storage vial and assessed for viability and level of contamination.

**Results:**

Stored liquid isolates demonstrated MTBC growth within an average time-to-detection of 18 days following retrieval, even at 21 months post storage. Contaminating organisms were detected in 2 of 80 (2.5%) culture isolates.

**Conclusion:**

Microbank^TM^ beads will allow for the reculture of up to 25 culture isolates using a reduced culture volume compared to current storage methods. Microbank^TM^ beads represent a storage solution for the medium-term storage of MTBC isolates.

**What this study adds:**

This study evaluated the use of Microbank^TM^ beads as an alternate method for storing MTBC culture isolates at −70 °C and provided a suitable option for medium-term storage of MTBC.

## Introduction

Tuberculosis diagnosis is a challenge in many health centres. The introduction and subsequent endorsement by the World Health Organization of the GenoType MTBDR*plus* (HAIN Lifesciences, Nehren, Germany),^1^ Xpert MTB/RIF and Xpert MTB/RIF Ultra assays’ (Cepheid, Sunnyvale, California, United States),^2,3^ and the Truenat MTB, MTB Plus and MTB-RIF Dx assays (Molbio Diagnostics, Goa, India)^4^ highlighted the advantages of using molecular technologies for diagnosis. Although there is a lag in developing new tuberculosis diagnostic assays, in July 2021, another group of technologies received the World Health Organization’s conditional recommendation for use in tuberculosis diagnosis.^5^ These are cobas MTB and cobas MTB-RIF/INH (Roche, Basel, Switzerland),^6^ Fluorotype^®^ MTBDR (Bruker, Nehren, Germany),^7^ RealT*ime* MTB (Abbott, Illinois, United States),^8,9^ and the BD MAX MDR-TB assay (Becton Dickinson, Franklin, New Jersey, United States).^10^ The Treatment Action Group report provides information on current and new screening and triage tools.^11^ The expanding tuberculosis diagnostics pipeline and other non-molecular, culture-based, microscopy and radiology innovations can increase testing and earlier disease detection, even in difficult-to-detect cases, such as in HIV co-infected individuals; expand the spectrum of drug-susceptibility testing; and reduce costs.^12^ Once a technology becomes available, it must be validated to ensure conformity to the manufacturer’s claims and/or target product profiles and verified to assess suitability within the intended settings.^13,14,15,16,17,18^ Both validation and verification require suitable reference material that is stable and can be safely and efficiently transported. Mycobacterial species pose a potential biosafety risk, especially for staff responsible for storing and transporting or aliquoting isolates. Culture isolates should also be stored in an appropriate medium and at the correct temperature to maintain viability. These requirements have both cost and space implications.

One of the earliest studies on long-term storage and preservation of mycobacteria was performed in 1972.^19^ *Mycobacterium tuberculosis* complex (MTBC) (cultured on Proskauer and Beck medium) and *Mycobacterium bovis* (cultured on Sauton’s medium) were stored in nine different diluents. These included sterile skim milk; 1% gelatin buffered at pH 6.8; 1:5 dilution of Sauton’s medium; 0.25% Triton WR 1339 in Sauton’s medium; a solution containing 8.3% dextran, 7.5% glucose, and 0.025% Triton WVR 1339; 15% aqueous solution of lactose at pH 5.0; 5% sodium glutamate; Tween-albumin medium; and distilled water. Archive stocks were stored at two different temperatures (−20 °C and −70 °C) for 3 years. There was a significant reduction in MTBC isolate viability when stored at −20 °C versus −70 °C. In addition, the methods applied had safety concerns, such as the bottling procedures, appropriate container selection, transportation, and the risk of aerosolisation (once the bottle was opened). Although most of these issues were resolved, the storage media and safety considerations were not ideal.^19^

In 2005, Huang et al. demonstrated that MTBC isolates from solid media could be stored at −70 °C in 7H9 broth without significant loss in viability (> 90%) for up 7 years, while the viability of strains preserved directly from mycobacteria growth indicator tubes (MGITs) was acceptable for up to 3 years.^20^ In 2014, the World Health Organization released a laboratory manual recommending MTBC isolate storage in 7H9 broth with glycerol,^21^ media preparation, specific equipment, and sterile work areas. Although the World Health Organization-recommended technique efficiently stores and maintains MTBC strain viability, Metcalfe et al. and Hanekom et al. reported a possible loss of strain fitness due to the subsequent need for sub-culture to maintain stock culture isolates.^22,23^ Furthermore, subculturing is prone to contamination, which may further risk the loss of valuable isolates. Kremer et al. reported an alternative option to collect samples from the surface of a frozen medium by scraping the top layer without defrosting the remaining suspension.^24^ However, the use of a frozen stock may reduce the viability of isolates if the entire tube thaws during the subculturing process. Glass beads have demonstrated medium-term storage for MTBC with strain recovery rates of > 94% within 30 days of incubation by Giampaglia et al.^25^ However, both the beads and storage vial require pre-sterilisation and preparation of storage medium, which increases the process cost and is labour intensive.

The Microbank^TM^ platform (www.https://pro-lab.com/products/clinical-microbiology/bacteriology/microbank/) has approximately 25 sterile beads per storage vial and a specifically formulated cryopreservative for low-temperature storage. It is potentially a suitable alternative storage solution for MTBC isolates. The Microbank^TM^ beads are porous, allowing microorganisms to adhere to the bead surface. A new culture can be grown by inoculating culture media with a single bead from the storage. Thus, the process can be repeated for the 25 beads of each isolate compared to the current method, where the number of possible new cultures depends on how much of the stock is used for each reculture. Some studies^26,27,28^ have reported reasonable fungal isolate recovery rates (96% – 100%) from the Microbank^TM^ beads; however, as far as we know, the Microbank^TM^ storage solution has not been assessed for MTBC isolate recovery. Nevertheless, the Microbank^TM^ beads may maintain longer MTBC viability and allow maximum usage with 25 beads, reducing process cost and preserving precious culture isolates.

This study aimed to determine the feasibility of using the Microbank^TM^ beads to store MTBC isolates from a laboratory in South Africa.

## Methods

### Ethical considerations

Ethics approval to store MTBC isolates from residual MTBC-positive liquid culture on the Microbank^TM^ system was obtained from the Human Research Ethics Committee at the University of the Witwatersrand in South Africa (ethics approval number: M1511110). The MGIT culture isolates were allocated a study number to maintain participant confidentiality. Informed consent was, therefore, not required. Isolates were described based solely on their laboratory characteristics, including MGIT Ziehl Neelsen, blood agar, line probe assay and drug-susceptibility testing.

### Preparation of culture isolates for Microbank^TM^ bead storage

Twenty residual MTBC-positive culture isolates from the Department of Molecular Medicine and Hematology research laboratory at a university in South Africa, containing a heavy initial inoculum (approximately 10^5^ to 10^6^ colony-forming units per millilitre [CFU/mL]) from MGITs were selected. The time-to-detection (TTD) for each culture isolate was determined and provided by the BACTEC™ MGIT 960 Mycobacteria Culture System (Becton Dickinson, Sparks, Maryland, United States) up to 42 days after incubation. Isolates comprised rifampicin- and isoniazid-susceptible, multidrug-resistant, and rifampicin-only or isoniazid-only resistant strains. An ATCC 25177 strain (Davies Diagnostics, Johannesburg, South Africa) was included as a positive control. Before their storage on the Microbank^TM^ beads (Pro Lab Diagnostics, Texas, United States) in February 2020, the liquid culture isolates were stored in their original tubes at room temperature for ~5 months following removal from the BACTEC™ MGIT 960 System. The MGITs were vortexed for 30 s to break up clumps before removing 1 mL of liquid culture. The bead inoculum was prepared by centrifuging 1 mL of liquid culture at 3000 *g* for 15 min at room temperature. A volume of 500 μL of supernatant was removed, and the remaining 500 μL of the re-suspended pellet (using a pipette) was added to one Microbank^TM^ bead vial. The resulting suspension was then inverted 10 times and incubated at room temperature for 60 min allowing the mycobacteria to adhere to the bead surface. The liquid was then aspirated, and the vial was stored at −70 °C per manufacturer’s instructions.^29^ At defined time points (16 months [15 June 2021] and 21 months [18 November 2021]), two beads were aseptically removed with a pipette tip from each isolate vial. The beads were then added to two separate MGITs containing 0.8 mL of polymyxin B, amphotericin B, nalidixic acid, trimethoprim and azlocillin antibiotic mixture (Becton Dickinson, Sparks, Maryland, United States) and incubated in a BACTEC™ MGIT 960 System at 37 °C for a maximum of 42 days. For those culture isolates that flagged positive, the presence of acid-fast bacilli was confirmed by smear microscopy (Ziehl Neelsen stain).^30^ The presence of contaminating organisms were confirmed by visual inspection of any ‘abnormal’ growth in the MGITs (where possible) and/or plating an aliquot of the culture onto blood agar and then analysing the plate for any growth after 48 h of incubation at 37 °C. A MGIT TBc Identification MPT64 antigen test (Becton Dickinson, Sparks, Maryland, United States) test was used to confirm the presence of MTBC in the MGIT isolates. Staff performing the storage and retrieval procedure were asked to compare the Microbank^TM^ procedure (in terms of ease to use) to the glycerol procedure currently used in the laboratory.

### Data analysis

The MGIT TTD for each bead was obtained from the BACTEC™ MGIT 960 printout. The TTDs were captured on a Microsoft Excel spreadsheet (Microsoft Corporation, Redmond, Washington, United States). A one-way analysis of variance was performed on the culture isolate TTD using SAS (SAS Institute Inc 2013. SAS/ACCESS^®^ 7.1 Interface to ADABAS, Cary, North Carolina, United States) to determine if there were significant differences at each time point. In addition, the number of archive beads that successfully grew MTBC, the number contaminated, and the number that failed to grow were reported.

## Results

**FIGURE 1:**
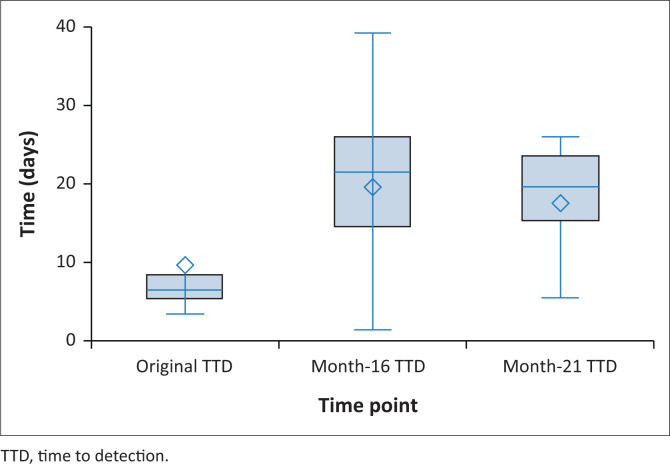
Distribution of means for the original, month 16 (15 June 2021), and month 21 (18 November 2021) time to detection at a laboratory in South Africa. The upper and lower ends of the box are the upper and lower quartiles. The box covers the interquartile interval, where 50% of the TTDs are found. The diamond represents the mean TTD at each timepoint and the horizontal line in each box represents the median TTD at each timepoint.

After a storage period of 16 and 21 months, 34 of 40 (85%) archive beads successfully grew MTBC, 5 of 40 (12.5%) did not demonstrate growth after the 42-day incubation, and 1 of 40 (2.5%) demonstrated contamination (Figure 1). The baseline TTD for the positive control culture isolate, ATCC 25177, was 15.9 days (Table 1). The positive control demonstrated a mean TTD of 8.4 days and 20.5 days after 16 months and 21 months. For isolates, where both beads retrieved at the same time point, demonstrated growth of MTBC, 15 of 16 and 13 of 15 after 16 and 21 months showed similar TTDs (Figure 1). Isolates 4 and 17 demonstrated > 8-day difference in TTD between the two beads retrieved simultaneously. The box and whisker plot (Figure 1) displays the distribution of means representing the TTD for the original culture, month 16 and month 21. The one-way analysis of variance indicated a significant difference among the means of the original, month 16 and month 21 TTD (F (2, 57) = 9.17, *p* < 0.001) (Online Supplementary file Table 1). Post-hoc analysis using Tukey’s Honestly Significant Difference test revealed that the original TTD (mean = 8.06 days) was significantly lower than both month 16 (mean = 19.18 days) and month 21 (mean = 17.10 days) TTD. However, no significant difference was observed between the mean culture isolate TTD at month 16 and month 21.

**TABLE 1:** Time to detection for culture isolates retrieved from Microbank^TM^ beads at two sampling points, 16 (15 June 2021) and 21 months (18 November 2021) at a laboratory in South Africa.

Culture isolate number	LPA result			Original TTD†	16-month storage		21-month storage	
	MTBC	Rifampicin resistance	Isoniazid resistance		Bead 1 TTD	Bead 2 TTD	Bead 3 TTD	Bead 4 TTD
1	D	ND	ND	3.16	22.04	21.19	25.16	23.22
2	D	ND	D	3.17	5.40	4.21	4.21	4.19
3	D	ND	D	3.18	23.14	24.11	18.12	20.10
4	D	ND	D	4.22	27.90	13.19	27.30	19.16
5	D	ND	ND	4.23	4.30	4.19	4.14	4.70
6	D	D	D	4.70	15.23	14.20	15.00	16.70
7	D	D	D	5.22	27.10	27.30	26.70	24.21
8	D	ND	D	7.18	23.12	23.17	23.50	22.22
9	D	D	D	7.23	18.70	20.16	20.16	17.17
10	D	ND	ND	11.70	20.90	21.14	18.13	17.10
11	D	ND	ND	17.00	12.10	13.23	11.23	13.17
12	D	ND	ND	17.20	16.16	16.19	15.00	12.60
13	D	ND	ND	5.11	24.40	22.20	20.20	n/a
14	D	D	ND	4.20	38.60	32.11	n/a	23.70
15	D	ND	ND	7.15	n/a	19.08	24.60	25.90
16	D	D	D	36.00	27.12	28.60	n/a	n/a
17	D	ND	ND	7.12	n/a	n/a	15.10	25.00
18	D	ND	D	2.10	n/a	n/a	20.18	n/a
19	D	ND	D	5.16	40.18‡	39.21	15.11	15.17
20	D	D	D	6.17	29.12	28.10	15.90	21.18‡
ATCC 25177	D	ND	ND	15.90	12.14	4.60	19.30	21.70

MTBC, *Mycobacterium tuberculosis* complex; TTD, time to detection (reported in days and hours); LPA, line probe assay; D, detected; ND, not detected; n/a, not applicable since no growth was observed; ATCC, American Type Culture Collection.

† , Time to detection and reported by the MGIT^TM^ 960 System for the original culture (prior to inoculation on the microbeads).

‡ , Contaminated culture isolate.

Regarding usability, laboratory staff reported that the Microbank^TM^ beads were technically undemanding (requiring only routine laboratory procedures such as centrifugation) and easy to use. Furthermore, no additional preparation of reagents was required.

## Discussion

Storage of MTBC culture isolates on the Microbank^TM^ beads was performed at −70 °C as recommended^19,21,31^ and MTBC isolates were recovered within 39 days of incubation in the BACTEC™ MGIT™ 960 instrument with minimal contamination. For archive cultures where contamination was seen, culture isolates grown from the second bead from the same vial did not show contamination suggesting that contamination occurred during the MGIT reculture process rather than during processing for storage. Since the Microbank^TM^ bead platform doesn’t address the possible contamination problem due to multiple openings and closing of the vials, sterility during the reculture process is critical.

Although the statistical analysis showed a significant difference in the TTD between the original and post-storage TTD, the TTD between the 16-month and 21-month time points was not statistically significant. This increased TTD could be attributed to decreased metabolic activities of the MTBC bacilli due to their storage at low temperatures.^31^ There was variability in TTD at both time points post storage, even in beads from the same vial, which may suggest that growth depends on the number of bacilli adhering to the bead. Rather than a loss in viability, a decreased number of bacilli on some beads may also be the reason for some culture isolates demonstrating a lack of growth after the 42-day incubation period and highlighting the importance of the uniform distribution of bacteria. It is therefore essential to rigorously invert the Microbank^TM^ vial at the time of inoculation to assist with the uniform distribution of the bacteria across the beads. The TTD has been described as a time-to-event variable, which represents an indirect measurement of the bacterial load,^32^ and it is possible to reduce the TTD by increasing the colony-forming units of the initial inoculum. Increasing colony-forming units may be achieved by incubating the culture isolates at 37 °C for a more extended period to allow for increased growth or increasing the starting volume of the inoculum. The susceptibility profile of the culture isolates also did not appear to impact the viability of the culture isolate or the TTD.

Recovery rates of MTBC preserved from culture media (stored at −70 °C for 7 years) can vary from 97.4% to 100%.^20^ Room temperature storage of culture isolates has also been investigated.^33^ Although the bacteria remained viable for up to 6 years, recovery rates were low (54.0% to 66.4% depending on storage conditions, such as the availability of an air conditioner) with a contamination rate of ~7%. Recovery rates of MTBC using the microbead method after a 21-month storage period were demonstrated to be 85%. Although this lower recovery rate appears to be a limitation of this method, further investigation is required using an increased inoculum volume or a higher culture concentration and a longer storage period.

A key advantage of the Microbank™ beads is their safety profile. The minimal liquid volume reduces the risk of leakage and subsequent aerosolisation. The system also does not require the preparation and autoclaving of media. Due to the increased risk of defrosting the entire vial and potential loss of the stock culture isolate, current practice recommends storing two vials of stock material in two separate freezers, which already has cost and space implications.^34^ With the Microbank™ bead storage, 25 individual isolate recoveries can be made from a single vial, allowing efficient storage, which is ideal for space-constrained environments. With routine subcultures, it has been reported that frequent subcultures may distort drug-susceptibility levels and patterns of strains,^35^ but with the Microbank™ system, since the primary culture isolate is technically not sub-cultured, this risk is minimal. For ease of identification within biorepository settings, Microbank^TM^ beads are available in a variety of colours.

Although not investigated in this study, Microbank^TM^ beads provide an attractive alternative for transporting viable isolates and for applications such as proficiency testing. The current transport of infectious MTBC culture isolates for further confirmatory reflex testing, such as sequencing, requires strict safety adherence criteria and is costly. Typically a secondary watertight container with absorbent material, together with outer packaging,^36^ is employed for shipping isolates and limitations are placed on the total allowable volume of liquid material in a single shipping container.^37^ The Microbank^TM^ bead platform will require the same safety considerations as conventional storage but offers a reduced risk of aerosolisation since the beads are not transported with any liquid.

### Limitations

This study has some limitations, the most important being the absence of long-term storage data and a small sample size. Investigation into the use of Microbank^TM^ beads for transporting MTBC was not performed.

### Conclusion

The use of Microbank^TM^ beads for medium-term storage of MTBC isolates at −70 °C shows promise, with growth observed within ~5 weeks of resuscitation, even at 21 months sampling post storage, and demonstrates advantages in ease of use.
